# Quantification of urinary total luteinizing hormone immunoreactivity may improve the prediction of ovulation time

**DOI:** 10.3389/fendo.2022.903831

**Published:** 2022-10-05

**Authors:** And Demir, Matti Hero, Elina Holopainen, Anders Juul

**Affiliations:** ^1^ New Childrenʼs Hospital, Pediatric Research Center, University of Helsinki and Helsinki University Hospital, Helsinki, Finland; ^2^ Department of Obstetrics and Gynecology, University of Helsinki and Helsinki University Hospital, Helsinki, Finland; ^3^ Department of Growth and Reproduction, Copenhagen University – Rigshospitalet, Copenhagen, Denmark; ^4^ Department of Clinical Medicine, University of Copenhagen, Copenhagen, Denmark

**Keywords:** Luteinizing hormone, LH-beta, LH core fragment, estrone-3-glucuronide, E3G, ovulation predictor kit, urine, women

## Abstract

**Objectives:**

Most of the currently available ovulation prediction kits provide a relatively rough estimation of ovulation time with a short fertility window. This is due to their focus on the maximum probability of conception occurring one day before ovulation, with no follow-up after LH surge until ovulation nor during the subsequent days thereafter. Earlier studies have shown that urine of reproductive age women contains at least 3 different molecular forms of luteinizing hormone (LH); 1) intact LH, 2) LH beta-subunit (LHβ) and a 3) small molecular weight fragment of LHβ, LHβ core fragment (LHβcf). The proportion of these LH forms in urine varies remarkably during the menstrual cycle, particularly in relation to the mid-cycle LH surge. In this exploratory study, we studied the potential implications of determining the periovulatory course of total LH immunoreactivity in urine (U-LH-ir) and intact LH immunoreactivity in serum (S-LH-ir) in the evaluation of the fertility window from a broader aspect with emphasis on the post-surge segment.

**Methods:**

We determined total U-LH-ir in addition to intact S-LH-ir, follicle-stimulating hormone (FSH), progesterone, and estradiol in 32 consecutive samples collected daily from 10 women at reproductive age. Inference to the non-intact U-LH-ir levels was made by calculating the proportion of total U-LH-ir to intact S-LH-ir.

**Results:**

Total U-LH-ir increased along with LH surge and remained at statistically significantly higher levels than those in serum for 5 consecutive days after the surge in S-LH-ir. S-LH-ir returned to follicular phase levels immediately on the following day after the LH surge, whereas the same took 7 days for total U-LH-ir.

**Conclusions:**

The current exploratory study provides preliminary evidence of the fact that U-LH-ir derived from degradation products of LH remains detectable at peak levels from the LH surge until ovulation and further during the early postovulatory period of fecundability. Thus, non-intact (or total) U-LH-ir appears to be a promising marker in the evaluation of the post-surge segment of the fertility window. Future studies are needed to unravel if this method can improve the prediction of ovulation time and higher rates of fecundability in both natural and assisted conception.

## Introduction

In order to optimize the probability of conception in a menstrual cycle, the appropriate timing of intercourse is of utmost importance. Randomized controlled trials show evidence that ovulation predictor kits (OPKs) may increase pregnancy rates (1).

In ovulatory cycles, ovulation usually occurs about 14 days before the onset of the next period. The length of the normal ovulatory cycle may vary considerably (26-35 days, mean 28 days), and extensive variations both in follicular (10-23 days) and luteal phases (7-19 days) have been reported (2, 3). Thus, making the prediction of ovulation and appropriate timing for intercourse or natural cycle intrauterine insemination is rather challenging (2, 4).

Since the ovulation time may vary from cycle to cycle, women are required to apply a urine test daily from the mid-follicular phase until getting a positive result, which causes undue stress in addition to financial burden (5, 6). The majority of currently commercially available OPKs accurately detect the urinary LH (U-LH) surge, which gives only a rough estimate of imminent ovulation. The LH surge occurs roughly 1 or 2 days prior to ovulation (7, 8). The maximum probability of conception in intercourse is one day before ovulation. If testing is performed after the LH peak has taken place due to various reasons, such as personal reasons or variations in the expected duration of the follicular phase, ovulation can be missed. Also, the vast majority of ejaculated spermatozoa remains viable in the female reproductive tract for 3-5 days (9), and an ovum can be fertilized usually for 24 hours after ovulation (10). Thus, there is a need to cover the early postovulatory segment of the fertility window to improve the currently available OPKs.

We recently demonstrated the occurrence of three distinct forms of LH immunoreactivity (LH-ir), i.e. intact LH and its degradation products, namely LH beta-subunit (LHβ), and a 12 kD fragment of LHβ, called core fragment (LHβcf) by a commercially available diagnostic method in urine samples obtained from fertile women (11). The proportion of these distinct forms of urinary LH-ir (U-LH-ir) varied significantly during the periovulatory period and total U-LH-ir prevailed for at least 3 days following the day of LH surge (12). Based on the findings of our recent studies (11, 12), we hypothesized that evaluation of the periovulatory course of different forms of U-LH-ir may provide valuable information about the post-surge segment of the fertility window.

In this exploratory study, we investigated the potential use of total U-LH-ir measurements along with S-LH-ir, serum estradiol and progesterone determinations for the evaluation of a broader fertility window beyond the LH surge, which may eventually improve the prediction of ovulation time and fecundability.

## Materials and methods

### Subjects

This study was conducted at the Department of Growth and Reproduction, Copenhagen University Rigshospitalet, Denmark and the Children’s Hospital, University of Helsinki, Finland. Ten healthy women (aged 18 to 40 years) visiting the former hospital volunteered to participate in the study. Inclusion criteria included being a healthy woman in the reproductive age range. Any history of irregularity in menstrual cycles was an exclusion criterion. Exclusion criteria also required that none of the subjects had a history of endocrine or metabolic disease and none were using any medication or hormonal contraceptives known to interfere with reproductive function at the time of the study. All the subjects had regular menstrual cycles (length of cycle 29.9 ± 5.1 days, duration of menstrual flow 5.6 ± 0.8 days; both expressed as mean ± 2 SD), and they were prospectively enrolled in the study with due consent. The study protocol was approved by the ethics committee of Copenhagen University Rigshospitalet. Laboratory investigations of the samples obtained from the subjects were performed in both institutions.

### Study design

Blood and urine samples were collected every morning at 8:00 am for 32 consecutive days. Every second day the subjects fasted overnight before blood sampling. The day of ovulation was determined in reference to the day of peak serum follicle-stimulating hormone (FSH) and luteinizing hormone (LH) levels. For each participant, the 32 consecutive days were transformed into days in each individual cycle, based on the data from the 3 consecutive menstrual cycles prior to initiation of the study. The regularity of the menstrual cycles in each individual was hence confirmed by a 3-month registration of menstrual bleedings (without blood and urine sampling). Urine was collected every morning except during menstrual flow and stored at +4°C for up to 10 days (2-3 days on average) before analysis. The term “LH surge” referred to the surge in LH-ir in serum (S-LH-ir).

### Assays

The immunofluorometric assays (IFMA) utilized in this study are commercially available sandwich assays using monoclonal antibodies (AutoDELFIA hFSH and hLH [the latter formerly known as LHspec], Wallac, PerkinElmer Finland Oy). One antibody is immobilized onto a microtiter strip well and the other one is labeled with a europium chelate. Both the capture and the detection antibody are directed toward the ß-subunit of LH recognizing different, distinct epitopes (13). This LH assay which has been designed specifically to detect intact LH and LHβ, but not human chorionic gonadotropin, measured also LHβcf as shown in our earlier study (11). Therefore, h-LH assay in this study measured total U-LH-ir, deriving from the intact LH, LHβ, and LHβcf. However, the serum LH (S-LH) assay measured only intact S-LH-ir, because LHβ and LHβcf concentrations were at negligible levels in serum (12). Due to the unavailability of a different assay for detecting intact U-LH-ir in this study, the non-intact LH-ir could not be determined as the arithmetic difference between total and intact LH-ir as performed in our previous studies (11, 12). Therefore, inference to the non-intact U-LH-ir levels was made by calculating the proportion of total U-LH-ir to intact S-LH-ir (**Figure 2**) because of the high correlation between U-LH-ir and S-LH-ir at similar absolute concentrations as shown in our earlier studies (14–16). The assays were performed according to the instructions of the manufacturer. A sample volume of 25 µL was used for serum and urine. The total assay volume was 225 µL. The assays were calibrated against the WHO Second International Standard for pituitary LH for immunoassay (80/552) and the Second International Reference Preparation of Pituitary FSH/LH (78/549), respectively. The limits of detection calculated by utilizing both the measured limits of blank and test replicates of a sample known to contain a low concentration of the analyte for the U-LH, U-FSH, S-LH, and S-FSH assays were 0.015 IU/L, 0.018 IU/L, 0.020 IU/L, and 0.035 IU/L, respectively (17). The intra- and inter-assay CVs for the U-FSH and U-LH assays ranged between 2.3% and 5.7%, and 5.2% and 6.4%, respectively (16). The intra-assay coefficients of variation for both assays were <2% at levels between 3 and 250 IU/l and about 10% at 0.3 IU/L. The inter-assay coefficient of variation was <3% at 4–18 IU/L for both FSH and LH (18). Hormone concentrations were not corrected for variations in urine excretion rate (such as urinary density or creatinine), because the correlation with serum levels was not improved but even impaired due to overcorrection in very dilute urine samples (14).

Serum samples were analyzed for progesterone and estradiol by RIA assays (Diagnostic Products Corporation, Los Angeles, USA and Immunodiagnostic System Ltd. Boldon, UK; respectively). For the progesterone assay, sensitivity was 0.23 nmol/L, and intra‐ and inter-assay CVs were 3.8% and 8.6%, respectively. For the estradiol assay, sensitivity was 18 pmol/L and intra‐ and inter-assay CVs were 7.5% and 8.4%, respectively.

### Statistics

The paired-samples t-test was used to analyze differences in the concentrations of LH in urine and serum from the same subjects on the same day, whereas the Kruskal-Wallis test was chosen for the nonparametric comparison of day-to-day variations of a hormone or ratio for analyzing the significance of change between consecutive days of the menstrual cycle. Pearson correlation coefficient was used for calculating correlations. This study was designed as an exploratory study with the aim of generating new hypotheses and therefore formal power calculations were not performed.

## Results

### Overall changes in hormone levels during periovulatory days

**Figure 1:**
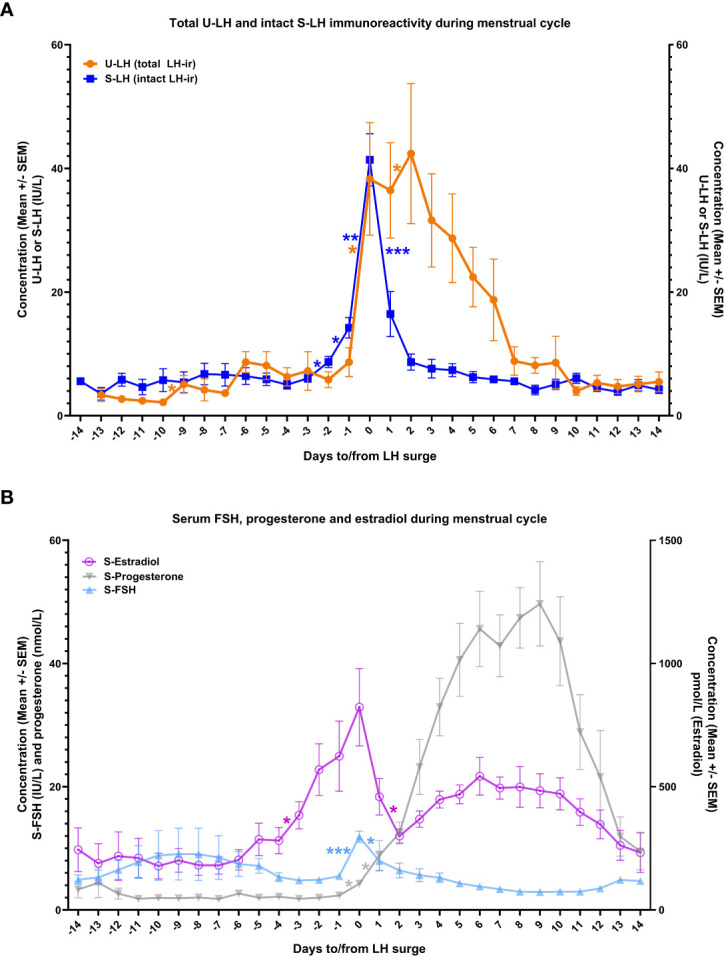
The course of total luteinizing hormone immunoreactivity (LH-ir) in urine and intact LH-ir in serum (total U-LH-ir and intact S-LH-ir, respectively) [panel **A**], and follicle-stimulating hormone (FSH), progesterone, and estradiol in serum [panel **B**] during the menstrual cycle. Symbols depict the mean values and bars represent the standard error of the mean. The statistically significant changes are denoted as follows: *P<.05, **P<.01, ***P<.001.

Normal changes in serum estradiol and progesterone levels confirmed the ovulatory cycles in this study population (**Figure 1B**). Serum LH concentrations increased steadily starting from day -3 onwards, with the steepest increase representing the LH surge on day 0, which was followed by a steep drop on day +1 (**Figure 1A**). These changes were associated with significant increases in serum progesterone concentrations continuously from day -1 through day +1 (**Figure 1B**).

There was no significant difference between the mean concentrations of S-LH and U-LH on day 0 (*P=*.74), indicating a similar pattern of increase in the concentrations of these two parameters on the day of LH surge. Also, serum FSH levels showed a similar pattern with an abrupt increase on day 0 followed by a drop on the following day; low serum FSH and high progesterone concentrations were maintained throughout the luteal phase (**Figure 1B**).

Urinary LH concentrations increased significantly again between days 1 and 2. Total U-LH-ir levels remained at significantly higher levels than those of S-LH-ir for 5 consecutive days following day 0 (*P<*.001). Unlike the steep fall in S-LH-ir levels right after the surge in S-LH-ir, the decrease in U-LH-ir was gradual over a one-week period following the LH surge. In contrast, S-LH-ir levels returned to follicular phase levels immediately on the following day after the LH surge and remained at similarly low levels thereafter with no significant day-to-day variations for at least 14 days after LH surge (**Figure 1A**).

**Figure 2:**
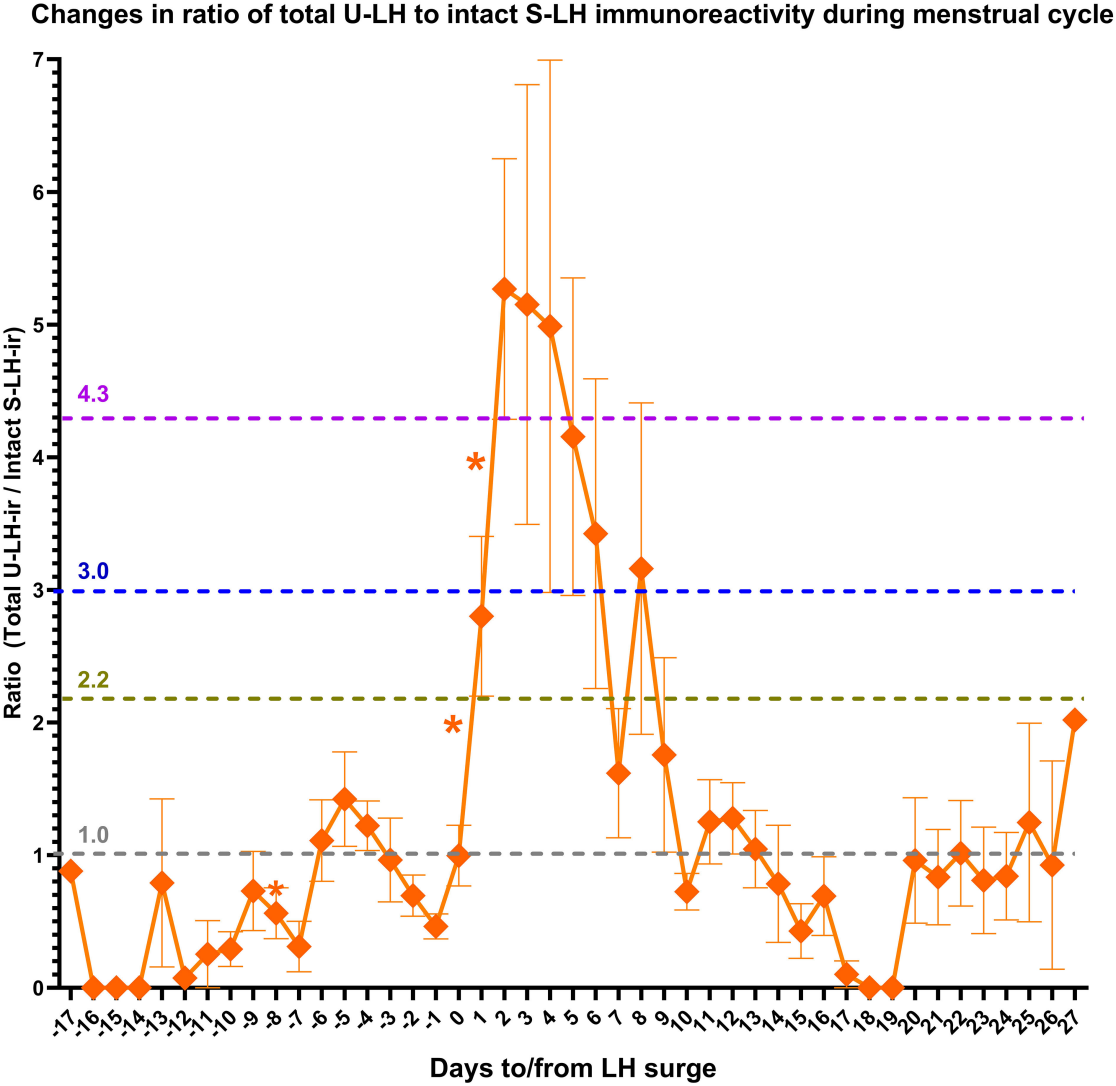
Changes in the ratio of total luteinizing hormone immunoreactivity (LH-ir) in urine to intact LH-ir in serum (representing the non-intact LH-ir in urine) during the menstrual cycle. Symbols depict the mean values and bars represent the standard error of the mean. The statistically significant changes are denoted as follows: *P<.05.

S-LH-ir levels started to increase already on day -1, causing a significant difference (*P=*.001) compared to U-LH-ir levels on the same day. The mean value of total U-LH-ir to S-LH-ir ratio was 1.0 on day 0 (**Figure 2**). Mean values of this ratio rose significantly over the next 2 days, 2 to 3-fold on day +1 and over 4 to 5-fold on day +2 (the former figure representing the fold increase shows the cautious estimate as the fold increases were calculated by considering not only the means but also the distributions of all the concentrations for each consecutive day). Likewise, the total U-LH-ir to S-LH-ir ratio did not fall below 3.0, 2.2, and 1 within 5, 6, and 9 days from the LH surge, respectively (**Figure 2**).

Serum estradiol concentrations measured on day -3 or -7 as well as integrated estradiol concentrations for the last 3 or 7 days before the LH surge correlated well with S-LH-ir levels on day 0 (**Table 1**). Serum estradiol concentrations measured on day -7 as well as integrated estradiol concentrations for the last 3 or 7 days before the LH surge correlated negatively with the U-LH-ir/S-LH-ir ratio on day 0, but not positively or negatively with that on day 1 (**Table 1**). Additionally, serum progesterone concentrations on day +1 (after significant increases on days 0 and +1, **Figure 1B**) correlated well with the U-LH-ir/S-LH-ir ratio during the period following the LH surge, very strongly during days 2-3, but not at all on day 0 (**Table 2**).

**Table 1:** Correlation between serum estradiol concentrations and S-LH concentrations or the ratio of total luteinizing hormone immunoreactivity (LH-ir) in urine to intact LH-ir in serum (representing the non-intact LH-ir in urine) around the days of LH peak.

	S-estradiol on day -3from LH peak	S-estradiol on day -7from LH peak	S-estradiol integrated for the last 3 days before LH peak	S-estradiol integrated for the last 7 days before LH peak
S-LH on day 0	0.78*	0.72*	0.70*	0.75*
U-LH/S-LH on day 0	0.54	-0.70*	-0.79*	0.78*
U-LH/S-LH on day +1	0.02	0.06	0.44	0.34

The statistically significant correlation (r) values are marked with an asterisk; *P<.05.

**Table 2:** Correlation of serum progesterone levels on the day after LH peak with the the ratio of total luteinizing hormone immunoreactivity (LH-ir) in urine to intact LH-ir in serum (representing the nonintact LH-ir in urine) during the first week after LH peak.

	U-LH/S-LH(day 0)	U-LH/S-LH(days 1-3)	U-LH/S-LH(days 2-3)	U-LH/S-LH(days 1-7)
S-progesterone (day + 1)	0.31	0.79*	0.85***	0.76*

The statistically significant correlation (r) values are marked with an asterisk; *P<.05, ***P<.001.

## Discussion

The period of high fertility prior to ovulation has previously been believed to include the five days prior to ovulation plus the day of ovulation (19–22). However, Wilcox et al. have revealed that these earlier assumptions are outdated in the light of several recent findings (23). According to this, the fertility window may extend for a much longer period, albeit with lower probabilities for conception, starting with a preovulatory period of up to 4 to 7 days and continuing with a postovulatory period of up to 2 days. Indeed, earlier studies revealed that the mean lifespans for sperm and ovum are 1.4 days and 0.7 days, respectively, and sperm would have a 5% probability of surviving more than 4.4 days and a 1% probability of surviving more than 6.8 days (9, 21, 24). Prediction of ovulation time is of crucial importance for timing the encounter of sperm and ovum within their lifespans for a conception with a reasonable probability.

Ovulation time may vary considerably even during 28-day-long regular cycles (23, 25, 26), making the prediction of LH surge and optimal fertilization time challenging. In efforts to overcome this challenge, a combination of different markers was studied (27–33). Studies published by WHO have demonstrated that the median time for a defined rise in the concentration of urinary E3G occurred on day 9 of the menstrual cycle, approximately 118 h (approximately 5 days) before the urinary LH peak in women with regular menstrual cycles (32, 33). Indeed, a rise in the concentration of E3G of 50% over the mean of the previous three values was shown to locate the start of the potentially fertile period (between day -3 and -7) in over 90% of the cycles (27, 28), thus a combination of urinary E3G and LH determinations has been used for the prediction of the optimal timing for conception (29–31, 34). On the other hand, current ovulation predictor kits (OPKs) do not detect any hormonal activity beyond LH surge for those who were “too late” and missed the day of LH surge, rather inform the ovulation time as a projection derived from the E3G and LH measurements from before the LH peak.

We recently demonstrated that urine from fertile women contains three forms of U-LH-ir, i.e. intact LH, LH beta-subunit (LHβ), and a 12 kD fragment of LHβ, called core fragment (LHβcf), the latter two forming the non-intact portion of LH-ir (11). The proportions of these forms vary remarkably during the menstrual cycle; non-intact LH-ir, particularly LHβcf is the major form of LH-ir for at least 3 days after the LH surge (11, 12). The LH-ir determined in this study was comprised of mainly intact S-LH-ir on the day of LH surge and of non-intact U-LH-ir during the post-surge days (**Figure 1A**), confirming the findings of earlier studies (11, 12).

The onset of the LH surge precedes ovulation by 35–44 hours, and the peak serum level of LH precedes ovulation by 10–12 hours (8, 35). This fact combined with the 24-hour fertilizability of an ovulated ovum (10) (and the 3-5 day viability of the ejaculated spermatozoa in the female reproductive tract (9)) indicates a window of fecundability for almost 3 days after the onset of the LH surge.

Findings of our earlier study had shown that the non-intact (degraded) portion of the U-LH-ir predicts the LH surge one day in advance and increases sharply after LH surge to five-fold until day +2 (hence until the day of ovulation) and remains over five-fold until day +3 and over three-fold until day +5 (12). All these phenomena were observed exactly at the same time points and magnitudes also in this study, further substantiating these findings as seen in **Figure 2**.

Other aspects including serum LH, FSH, progesterone, E2, inhibin A and B have previously been published (36, 37). **Table 2** shows that the non-intact U-LH-ir correlated strongly with the progesterone levels immediately before and after the ovulation but not any earlier (not on day 0), and at the strongest level on days 2 and 3 (which is the highest probable postovulatory period of time for conception).

Observations from our earlier studies (11, 12) also imply the possibility of utilizing data derived from the decreasing total (or non-intact) U-LH-ir before LH surge as an add-on to E3G in the algorithm if the urine sample was taken too close to an imminent LH surge or too late after an LH surge, because the fall in non-intact U-LH-ir and increase in E3G levels herald an impending LH surge within 1-3 days and 3-7 days in advance, respectively, but neither E3G nor LH surge data provide any predictive information in regard to the postovulatory segment of the fertility window.

S-LH-ir and U-LH-ir/S-LH-ir ratio on the day of LH surge correlated at similar levels positively and negatively, respectively, with the serum estradiol concentrations measured on day -7 as well as integrated estradiol concentrations for the last 3 or 7 days before LH surge (27, 28). The negative correlation between serum estradiol levels and U-LH-ir/S-LH-ir ratio disappeared on day +1.

Also, the significant fall in non-intact U-LH-ir on day -1 (represented by the total U-LH-ir/S-LH-ir ratio in this study) confirmed the findings of our earlier study (12). The falling trend in total U-LH-ir during days -4 through -1 further supports this finding (**Figure 2**). These findings together indicate the build-up of an intact LH pool until day -1, after which the trend reverses in favor of the non-intact U-LH-ir during the post-surge period for at least 5 days, the peak being observed on day +3 ( (12); also in **Figure 2**).

The current exploratory study provides preliminary evidence of the fact that U-LH-ir derived from degradation products of LH remains detectable at peak levels from the LH surge until ovulation and further during the early postovulatory period of fecundability. Thus, non-intact (or total) LH-ir appears to be a promising marker in the evaluation of the post-surge segment of the fertility window, which may improve the prediction of ovulation time and fecundability in both natural and assisted conception.

The current study design can be developed further by incorporating an assay that can detect intact U-LH-ir for assessing the total or non-intact to intact U-LH-ir ratio directly without further calculations. This limitation can be overcome by the availability of an assay to measure non-intact U-LH-ir or even better urinary LHβ and LHβcf concentrations separately. Unfortunately, non-intact U-LH-ir or its components cannot be measured directly at present due to the unavailability of antibodies specific for LHβ and LHβcf.

One other major reason behind the suboptimal functional utility of current OPKs is the variability of LH surge patterns. Park et al. and Direito et al. documented various examples of short, medium, double, and prolonged LH surges with single, double, multiple, or plateau peaks (10, 38). LH surges with several peaks were associated with statistically significant smaller follicle sizes before rupture and lower LH levels on the day of ovulation (10). Also, premature LH surges in women with regular menstrual cycles were reported (39). These all suggest that not all detected LH surges lead to ovulation even in regularly ovulating women. Some anovulatory events may be associated with false-positive results due to rises in LH, e.g., luteinized unruptured follicles, and hemorrhagic anovulatory follicles (40). False-positive results may also occur due to some OPKs of poor specificity detecting epitopes for intact LH, LHβ or LHβcf in the form of double, multiple, or plateau peaks (10, 38) rather than the targeted intact LH only, for which the product was designed to detect at the first place. On the other hand, there may be several missed cases in which signals of imminent ovulation remained undetected by current OPKs. Such false-negative results may be due to a failure to detect different naturally occurring LH variants (13, 41). The current OPKs with some or all of the above-mentioned drawbacks predict the LH surge by E3G and the ovulation by the LH surge, with no follow-up after the LH surge until ovulation nor during the subsequent hours thereafter.

We suggest that the utilization of highly specific intact LH assays designed for different LH variants to detect the true LH peak jointly with total (or non-intact) U-LH-ir assays may be combined with pre-surge E3G determinations for covering the postsurge segment of the fertility window. The findings of this study justify further research towards a novel OPK model, which could employ a more extensive ray of predictors for attaining a more accurate interpretation of the window of fertility as well as for distinguishing the LH surges of menstrual cycles with true ovulation from those without.

These preliminary findings yet lack clinical validation, thus meriting further research in the clinical setting for validating optimal test designs and algorithms, particularly due to the findings of the current study being based on 10 volunteers only. Another limitation was the lack of some exclusion criteria like the factor of alcohol consumption and aging of subjects, which may have induced alterations in the course of menstrual cycles or LH-ir, respectively. However, irregularity in menstrual cycles was an exclusion criterion for subjects in this study, therefore this limitation may be considered a minor one. On the other hand, the absence of ultrasound to confirm structural changes consistent with ovulation/anovulation was a major limitation of this study.

We conclude that future larger studies are needed to evaluate the utility of U-LH-ir levels by employing a gold standard test of ovulation, serum LH-ir and progesterone determinations along with the ultrasonographic evidence of ovulation. Such improved studies may unravel if a broader window of fertility could be achieved through the detection of periovulatory total LH-ir or its non-intact portion (LHβ or LHβcf) along with E3G concentrations in urine. This would mean improved algorithms for OPKs and higher rates of success in predicting ovulation time and attaining fecundability in both natural and assisted conception.

## Data availability statement

The raw data supporting the conclusions of this article will be made available by the authors, without undue reservation.

## Ethics statement

The studies involving human participants were reviewed and approved by Department of Growth and Reproduction, Copenhagen University - Rigshospitalet Copenhagen, Denmark. The patients/participants provided their written informed consent to participate in this study.

## Author contributions

All authors contributed to the article, accepted responsibility for the entire content of the manuscript and approved the submitted version.

## Funding

Part of the study was financed by grants from The Finnish Medical Foundation (Suomen Lääketieteen Säätiö).

## Acknowledgments

The authors would like to express their gratitude to the subjects for their cooperation in this study.

## Conflict of interest

The authors declare that the research was conducted in the absence of any commercial or financial relationships that could be construed as a potential conflict of interest.

## Publisher’s note

All claims expressed in this article are solely those of the authors and do not necessarily represent those of their affiliated organizations, or those of the publisher, the editors and the reviewers. Any product that may be evaluated in this article, or claim that may be made by its manufacturer, is not guaranteed or endorsed by the publisher.
